# Translational medications development for methamphetamine use disorder: A systematic review of preclinical, human laboratory, and clinical research

**DOI:** 10.1016/j.pharmr.2026.100145

**Published:** 2026-05-27

**Authors:** Alexandra N. Johansen, Mark A. Smith, William W. Stoops

**Affiliations:** 1 Department of Psychology, University of Kentucky College of Arts and Sciences, Lexington, Kentucky; 2 Department of Psychology and Program in Neuroscience, Davidson College, Davidson, North Carolina; 3 Department of Behavioral Science, University of Kentucky College of Medicine, Lexington, Kentucky; 4 Department of Psychiatry, University of Kentucky College of Medicine, Lexington, Kentucky

## Abstract

The prevalence of methamphetamine use disorder (MUD) remains discouragingly high. Relatively few clinical trials have focused on identifying new pharmacotherapies for MUD, and no medications have received US Food and Drug Administration approval. The lack of available pharmacotherapies may be due to failure to follow a rational, translational medications development pipeline progressing from preclinical work to the human laboratory to clinical trials. Our review thus has 2 primary goals: to (1) assess the scope of the literature evaluating candidate medications for MUD and (2) identify drugs screened to treat MUD across research domains, analyzing concordance across contexts. We identified 36 randomized, double-blind, placebo-controlled clinical trials that evaluated 25 candidate medications for MUD. Only 5 of these putative treatments (aripiprazole, bupropion, *d*-amphetamine, modafinil, and naltrexone) had also been evaluated in human laboratory and preclinical laboratory contexts. Overall, most studies showed no change in methamphetamine use (ie, no effects of treatment) across contexts. Although literature from these contexts imply a high degree of negative predictive validity, we encountered limitations at each level of analysis that prevented us from fully confirming concordance (eg, lack of positive predictive validity). These trends and limitations highlight the extent to which methamphetamine treatments are under-researched relative to other substance use disorders, such as cocaine use disorder. To address this gap in the literature, we advocate for future work that identifies therapeutic targets and, by consequence, classes of medications (repurposed or novel) to treat MUD. We conclude this review with additional comments about future research directions and treatment considerations.

**Significance statement:**

Investment in methamphetamine use disorder (MUD) medications development remains poor. To date, no pharmacotherapies have received US Food and Drug Administration (FDA) approval to treat MUD, and few candidate medications have been systematically evaluated using a translational medications development pipeline (eg, beginning with preclinical research and progressing to human laboratory research and clinical trials). Adhering to the translational pipeline while incorporating new FDA guidance, such as evaluating nonabstinence outcomes, may be useful in facilitating MUD medications development.

## I Introduction

### A Epidemiology of methamphetamine use disorder

According to the 2024 National Survey on Drug Use and Health, approximately 1.4 million people aged 12 years and older reported past-month methamphetamine use, and approximately 2.4 million people reported past-year methamphetamine use.^1^ From 2015 to 2019, there was a stark 10-fold increase in methamphetamine use disorder (MUD) among people who identified as Black, along with at least a 2-fold increase in MUD among people who identified as White or Hispanic.^2^ Over the past decade, methamphetamine-related mortality has risen drastically, increasing 50-fold between 1999 and 2021.^3^

In addition to mortality, methamphetamine use is associated with many physical and psychosocial harms.^4^, ^5^, ^6^, ^7^, ^8^, ^9^, ^10^ Common methamphetamine-related causes of death include seizures, arrhythmias, and respiratory failure.^11^ Broadly, methamphetamine use is associated with cardiotoxicity, immune system dysregulation, impaired cognition, dysregulated sleep, and adverse psychiatric outcomes.^4^, ^5^, ^6^, ^7^^,^^9^, ^10^, ^11^ People who use methamphetamine are also more likely to contract blood-borne viruses by engaging in risky sexual behaviors and sharing needles for intravenous drug use, which contributes to higher rates of HIV seroconversion especially among men who have sex with men.^11^ Despite these apparent harms, many questions remain about methamphetamine’s neurobiological effects and, by consequence, the optimal pharmacological profile for putative treatments.

### B Methamphetamine pharmacology: Monoamine systems

Methamphetamine is a central nervous system psychostimulant that amplifies monoamine (eg, dopamine, serotonin, and norepinephrine) signaling. In terms of mechanism of action, methamphetamine binds to the vesicular monoamine transporter 2 and reverses the transporter’s function, releasing monoamines (especially dopamine) from vesicles into the synapse. Methamphetamine acts as a dopaminergic and noradrenergic reuptake inhibitor, prolonging extracellular monoamine activity. Additionally, high doses of methamphetamine may function as a monoamine oxidase inhibitor, thereby delaying catecholamine degradation and further extending extracellular action.^4^^,^^12^, ^13^, ^14^, ^15^ Collectively, these actions increase dopaminergic and other monoaminergic signaling.

The neurotransmitter dopamine plays a crucial role in emotion, and movement, and reward-related behaviors.^16^ There are 5 metabotropic dopamine receptor subtypes (D_1_–D_5_) primarily expressed in the central nervous system; these receptors subtypes are divided into 2 families according to pharmacology, structural homology, and function: D_1_-like (D_1_ and D_5_) and D_2_-like (D_2_, D_3_, and D_4_) receptors. They have opposing effects—the former pairs with stimulatory G-protein binding domains, whereas the latter pairs with inhibitory G-protein binding domains.^17^, ^18^, ^19^ The relative density of each receptor subtype in the brain varies regionally^19^ (see other works for a more thorough regional focus^20^).

Although dopamine signaling in the mesolimbic pathway, which projects from the ventral tegmental area to the nucleus accumbens, primarily governs psychostimulants’ rewarding effects,^21^^,^^22^ other monoamines also play an important role in psychostimulants’ abuse liability.^23^ Further, monoaminergic circuits do not act in isolation; for example, mu opioid receptor systems influence D_1_ receptors’ expression,^24^ glutamatergic neurons regulate dopaminergic activity in the striatum,^25^ and GABAergic neurons may alter glutamatergic and dopaminergic activity alike.^23^ Nonetheless, most of the extant literature focuses on dopamine.

Rodent studies have highlighted the role of dopaminergic signaling in methamphetamine self-administration. When rats received D_1_^26^ and D_1_-like^27^ receptor antagonists, they reduced methamphetamine self-administration. Interestingly, dual partial D_1_ agonist and D_2_ antagonist treatment attenuated methamphetamine intake,^28^ although other work has indicated that methamphetamine self-administration may be more sensitive to D_1_ antagonism than D_2_ antagonism.^27^ Likewise, D_3_ receptor antagonists^29^^,^^30^ and mixed dopamine receptor antagonists^31^ have decreased methamphetamine self-administration in rodent models. Recent work suggested that vesicular monoamine transporter 2 inhibition attenuates methamphetamine-evoked striatal dopamine release and methamphetamine self-administration alike.^32^ Little is known about the role of other monoamines in methamphetamine self-administration.

Many studies have focused on methamphetamine’s neurotoxic effects and related neuroadaptations.^4^^,^^9^ A robust body of evidence suggests that chronic (eg, long-term, near-daily) methamphetamine use causes dopamine depletion along with enduring damage to dopaminergic circuitry across species.^33^, ^34^, ^35^, ^36^ High doses of methamphetamine resulted in dopaminergic nerve terminal degeneration with related deficits observed in the rat neostriatum and nucleus accumbens,^35^ along with striatal dopamine and serotonin depletion.^37^ After rodents had self-administered methamphetamine for 5 weeks and subsequently undergone 24 hours of withdrawal, assays showed lower D_1_ receptor densities in the nucleus accumbens shell, along with reduced levels of D_2_ receptors in the dorsal-medial substantia nigra zona compacta and ventral tegmental area. These effects were not observed in yoked peers who received saline or the same quantity of noncontingent methamphetamine.^38^ Moreover, rats that self-administered methamphetamine showed enduring biochemical changes, including reductions in dopamine, dopamine transporter (DAT), and tyrosine hydroxylase protein levels, in the striatum and cortex analogous to those observed postmortem among people who used methamphetamine.^33^

Clinically, chronic methamphetamine use has been associated with reductions in striatal DATs,^36^ and people abstaining from methamphetamine showed significant reductions in dopamine receptor availability^34^ along with 10% to 16% reductions in striatal DAT and vesicular monoamine transporter 2 binding potentials^39^ relative to people who had not used methamphetamine. However, periods of protracted abstinence (12–17 months) counteracted this reduction in DATs. Although DAT levels remained lower in people who reported past methamphetamine use compared with controls, this difference was not significant.^40^

Few studies have evaluated the role of other monoamines in methamphetamine self-administration; further, the effects of methamphetamine use on other monoamines has received little attention in comparison. Limited data from positron emission tomography imaging suggest that people who report long-term methamphetamine use may have lower serotonin transporter availability relative to controls; notably, transporter availability may be inversely associated with duration of methamphetamine use.^41^ However, no studies have characterized the effects of methamphetamine use on other monoaminergic (eg, noradrenergic) circuitry in people. Elucidating the neuropharmacology of methamphetamine’s reinforcing and neurotoxic effects is crucial to identify new therapeutic targets.

### C Shortage of treatment development research for stimulants

The field has focused disproportionately on nonstimulant substance use disorders (SUDs) medications development. An extensive literature exists describing medications development for alcohol and other SUDs.^42^^,^^43^ For example, 3 medications have received US Food and Drug Administration (FDA) approval to treat alcohol use disorder (disulfiram, acamprosate, and naltrexone),^44^ and 3 medications have received FDA approval to treat opioid use disorder (buprenorphine, methadone, and naltrexone); many of these medications are available in multiple formulations (eg, depot injections).^42^ A variety of options exist to treat tobacco use disorder, including nicotine replacement therapies, bupropion, and varenicline.^45^ No FDA-approved treatments exist for cocaine use disorder. Among the 10 candidate medications that have been assessed using the translational medications development pipeline, *d*-amphetamine has shown promise.^46^

To date, no medications have received FDA approval to treat MUD. Moreover, no pharmacotherapies have met the common clinical trial standard (eg, total methamphetamine abstinence), failing to provide compelling evidence to support further investigation. Likewise, most nonpharmacological interventions, such as behavioral activation, cognitive-behavioral therapy, and psychosocial interventions, have shown limited effectiveness in reducing methamphetamine use.^4^^,^^9^ In contrast, contingency management has been associated with increased abstinence although it has not been widely implemented.^47^

Although the FDA has identified complete abstinence as the primary endpoint of interest for SUD medications development, a growing movement has acknowledged that this benchmark may be unrealistic for stimulant disorders and thus has proposed that meaningful partial reductions in use may also serve as a viable endpoint.^46^^,^^48^, ^49^, ^50^, ^51^ It is possible that this paucity of candidate medications for MUD may stem from poor adherence to a rational, translational medications development pipeline progressing from preclinical studies to the human laboratory and clinical trials (Fig. 1). Thus, the aims of the present review are 2-fold: (1) to evaluate the current scope of the literature screening candidate medications for MUD and (2) to identify putative medications assessed for MUD treatment across clinical trials, the human laboratory, and preclinical laboratory settings, assessing concordance across research contexts.

**Fig. 1:**
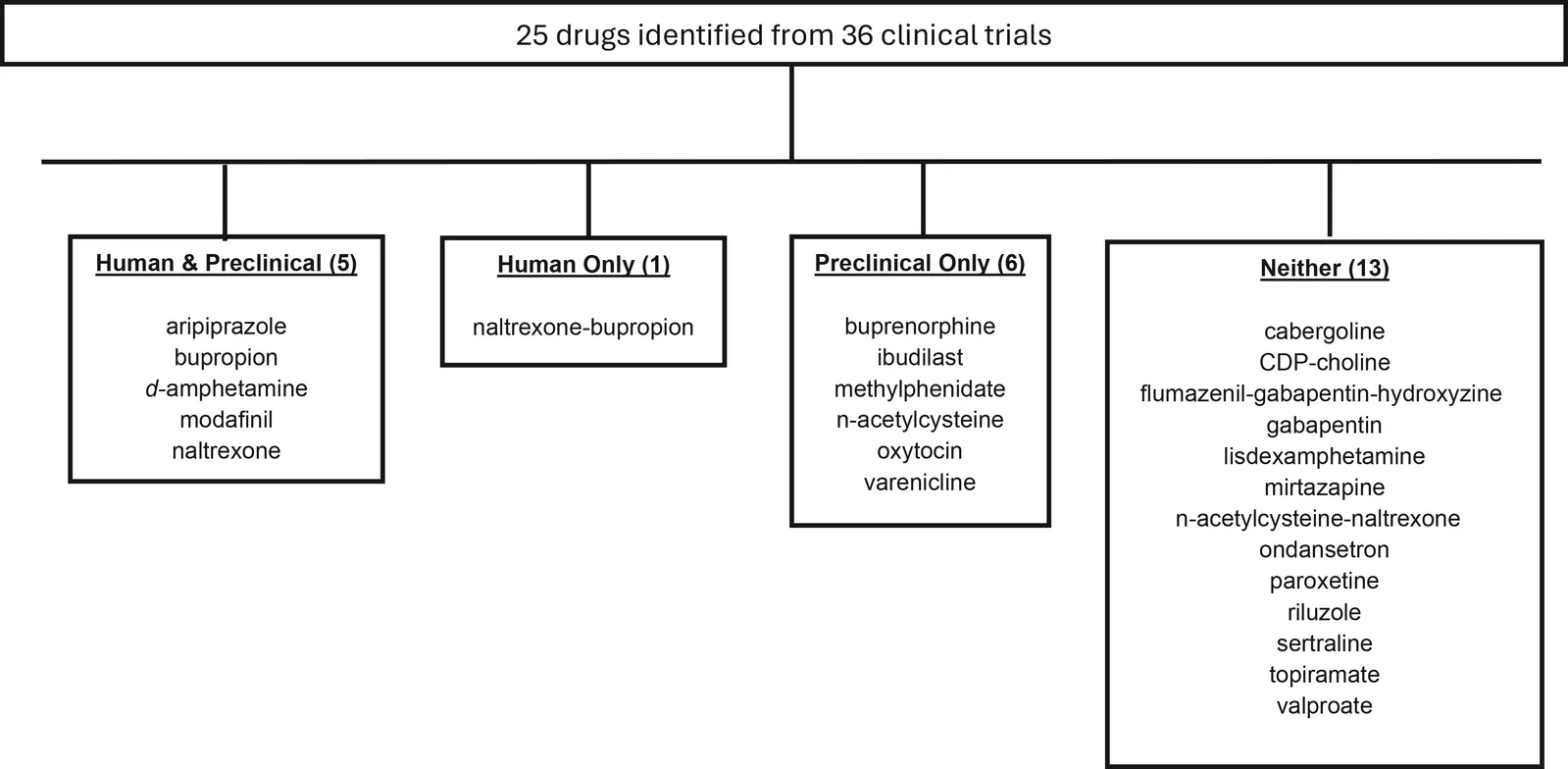
Diagram showing the putative medications identified from the searches in the present review. Medications are categorized based on the context(s) in which each had been evaluated: human and preclinical laboratory settings, human laboratory setting, preclinical setting, or neither.

## II Materials and methods

### A Review of common proxies to screen candidate medications

#### 1 Clinical trials

In the SUD medications development literature, common clinical trial outcomes of interest include program retention, self-report measures, and biological indicators of abstinence (eg, negative urine drug screens).^14^ Although program retention may be associated with treatment outcomes, the literature suggests that it is a weak indicator of treatment efficacy and overarching wellbeing post-treatment.^52^ Self-report measures, such as *frequency of use* reports obtained from the Timeline Follow Back method, serve as a convenient, inexpensive, and noninvasive way to measure substance use during treatment. However, their accuracy varies.^46^^,^^49^^,^^52^ As evidence suggests that people may under-report their substance use during treatment,^53^ self-report measures are often included in clinical trial data collection as a complement to more objective biological outcomes.^46^^,^^49^^,^^53^ To evaluate biological abstinence outcomes, clinical trials often use urine toxicology.^46^^,^^49^^,^^52^^,^^53^ Relative to other measures of clinical trial efficacy, urine drug screens are sensitive indicators of both pharmacological and behavioral treatment effects for stimulants.^52^ As others have described, assessing abstinence through urine toxicology requires biochemical analyses, wherein samples are tested for a specific cutoff. Results may be presented as the *percentage of negative urine specimens* or as a binary “*abstinent/non-abstinent*” outcome.^46^ Based on FDA guidance and standard practices in clinical trials for SUD medications development, the present review defines treatment efficacy as methamphetamine abstinence, based on qualitative urine drug testing results.

#### 2 Human laboratory studies

In the human laboratory, 2 complementary substance use indices appear frequently—subjective effects measures and drug self-administration procedures.^46^^,^^54^ Subjective reports collect both qualitative and quantitative data about the effects of contingent or noncontingent drug administration, as well as the absence of drug (eg, withdrawal and craving).^42^^,^^46^^,^^54^ It follows that these measures have often been used to assess drugs’ abuse liability.^46^^,^^54^^,^^55^ However, subjective effects scales indirectly measure drug use, often demonstrating insufficient predictive validity for self-administration outcomes.^54^^,^^56^ Subjective effects indices are more sensitive to pharmacological manipulations than drug self-administration,^57^ which contributes to the poor concordance between these 2 measures.^46^^,^^54^

In contrast, operant self-administration procedures directly measure drug use.^42^^,^^46^^,^^54^ Many reviews have discussed it extensively.^46^^,^^54^^,^^57^^,^^58^ In short, human laboratory self-administration procedures entail emitting a specified operant response (such as clicks on a computer mouse) to earn a quantity of drug. These procedures were originally developed in animal models and later adapted for the human laboratory.^54^^,^^58^, ^59^, ^60^, ^61^ Different contingencies may govern reinforcer delivery. Fixed-ratio (FR) schedules are commonly used in substance use research; under these conditions, participants must emit a set number of responses to obtain drug (such as an infusion of intravenous methamphetamine). For example, a person must press a button 5 times to earn a drug reinforcer on a FR5 schedule of reinforcement. For FR operant methamphetamine self-administration, the primary dependent variable is typically quantified as the total number of lever presses, drug intake, or number of drug infusions delivered over the course of the session. Drug self-administration studies may incorporate other types of intermittent schedules, such as fixed-interval, variable-ratio, or variable-interval schedules; under those contingencies, drug delivery may occur after a set amount of time (fixed-interval), variable number of operant responses (variable-ratio), or variable span of time (variable-interval).^54^

Although FR schedules of reinforcement measure level of responding for drug,^62^ progressive-ratio (PR) schedules of reinforcement measure motivation for drug.^54^^,^^63^ Under a PR contingency, the amount of effort (eg, number of responses or ratio value) required to obtain the same unit dose of drug systematically increases with each reinforcer obtained. The main outcome of interest is typically the “breakpoint,” the ratio value at which the subject no longer meets the schedule requirement. Generally, greater breakpoint values suggest greater abuse potential.

To improve ecological validity, some laboratory self-administration procedures provide a choice between a drug reinforcer and a nondrug reinforcer, such as money or food; these approaches better mirror the real-world choice and decision-making contexts in which substance use occurs.^64^^,^^65^ Distinct intermittent contingencies govern the delivery of each commodity, and procedures otherwise remain conceptually similar to traditional (single-commodity) operant self-administration. Notably, choice procedures measure not only drug use but also behavioral selectivity; in other words, they evaluate if pretreatments specifically target drug-taking behaviors or nondiscriminately affect other reinforcer-taking behaviors or overall motoric ability.^46^ Often, choice task outcomes are operationalized as indifference values, the ratio value at which behavior is allocated equally between available commodities.

Prior work has demonstrated good concordance between these human laboratory self-administration outcomes and clinical trial treatment efficacy, suggesting that drug self-administration is an effective way to screen putative pharmacotherapies for SUDs.^43^^,^^46^^,^^54^^,^^57^^,^^60^^,^^66^ Importantly, multiple pharmacotherapies originally screened in the human laboratory and later translated to the clinic effectively attenuated drug use in both contexts.^67^, ^68^, ^69^ Given the limitations of subjective reports, studies that solely measured methamphetamine’s subjective effects were not considered in this review. Consistent with the clinical trial inclusion criteria outlined above, we focused exclusively on studies that measured methamphetamine self-administration.

#### 3 Preclinical studies

As preclinical self-administration procedures were forward translated and adapted for human drug self-administration,^59^^,^^61^ they share many similar features. Both preclinical and human laboratory self-administration procedures use analogous reinforcement contingencies, task structures, and quantitative outcomes (lever presses, breakpoints, and indifference values). The predictive validity of preclinical self-administration may be assay-dependent^46^; for instance, dependent (long-term) models of drug use likely offer greater face validity than nondependent (sporadic or short-term) models of drug use.^66^ Although they offer lower predictive validity than human laboratory measures, preclinical self-administration proxies have moderate concordance with real-world outcomes, and they have aided in medications development for SUDs.^46^^,^^54^

For this review, we emphasize that preclinical drug self-administration refers to *drug-taking* behaviors, not *drug-seeking* behaviors (eg, extinction and reinstatement). Extinction refers to the repeated presentation of the conditioned stimulus (such as a cue light) without an unconditioned stimulus (drug). Subjects can emit operant responses during extinction, but the drug reinforcer is replaced with the drug’s vehicle (saline). Similarly, during reinstatement, subjects do not have response-contingent drug access. They instead receive a noncontingent drug-priming infusion, exposure to drug-related cues, or a stressor followed by extinction conditions (eg, vehicle access) for the remainder of the session. The presentation of these noncontingent stimuli causes a resurgence in drug-seeking behavior. It is important to note that these unreinforced responses do not occur in humans, which limits the face validity of these procedures. Additionally, as others^70^^,^^71^ have acknowledged, these phases of cessation and return to use are not voluntary, limiting translational appeal and predictive validity. Further, to date, no findings from rodent reinstatement models have contributed to the development of an FDA-approved pharmacotherapy for SUDs.^72^ Most importantly, neither extinction nor reinstatement measures active drug use.

As all clinical trials discussed in this review administered treatment to people who were actively using drugs (and not to those who were abstaining from drugs), extinction and reinstatement are incongruent with the present definition of treatment efficacy and fall beyond the scope of our manuscript. Moreover, medication effects on the acquisition of methamphetamine-reinforced responding in drug-naïve subjects is more commonly viewed as a model of prevention than treatment; consequently, those studies were also excluded from this review. Adhering to the FDA guidelines regarding new medications development for SUDs, results from preclinical self-administration studies (eg, drug-taking behaviors) remained the primary outcome of interest for this review.

In sum, the primary outcome of interest in this review is drug-taking behavior. As outlined below, we omitted studies that focused solely on measures of drug-seeking behaviors (eg, craving, subjective effects, extinction, or reinstatement) and the acquisition of responding in drug-naïve subjects. Instead, we only discuss studies with the following features that evaluated a putative methamphetamine pharmacotherapy: clinical trials with urine drug test results; human laboratory studies with measures of methamphetamine self-administration; and preclinical studies with measures of methamphetamine self-administration in drug-experienced subjects.

### B Study selection

All of the study selection procedures below abided by Preferred Reporting Items for Systematic Reviews and Meta-Analyses guidelines.^73^ In the present systematic review, we first used PubMed to identify blinded, randomized, placebo-controlled clinical trials that evaluated the effects of candidate pharmacotherapies on MUD or methamphetamine dependence. To qualify, studies had to include urinalysis as a measure of methamphetamine use. Further, studies that recruited participants who reported any comorbid SUDs (besides alcohol, nicotine, and cannabis) or comorbid psychiatric disorders were removed. Given these criteria, we selected the search terms “methamphetamine,” “clinical trial,” “randomized,” “blind,” and “placebo,” all connected by the Boolean operator AND. This search produced 151 records (pre-June 2025). After screening these records’ titles and abstracts, we excluded 79 papers identified as different study types (eg, reviews, secondary analyses, and clinical trial protocols, along with papers using nonhuman animals, other experimental designs, or different research settings). We then retrieved and assessed the remaining 72 reports; 36 reports did not meet the eligibility criteria. Namely, 28 reports did not use urinalysis to measure drug use, 7 reports included participants with comorbid disorders (eg, opioid/amphetamine co-use or psychiatric conditions, including bipolar disorder and bipolar depression), and 1 study was excluded for recruiting participants who were not dependent on methamphetamine. This initial search yielded 36 papers with 25 putative medications screened in clinical trials. Once we determined which candidate treatments had been assessed across all 3 research contexts, we excluded an additional 25 studies, which resulted in 11 trials included in this review (Supplemental Fig. 1A).

After evaluating the clinical trials, we used a similar approach to identify studies that analyzed the identified potential pharmacotherapies for methamphetamine use in the human laboratory. Studies had to include some measure of methamphetamine self-administration (FR, PR, choice, etc), a placebo control, and at least 1 of the medications identified through our clinical trials search. To this end, we used the search terms “methamphetamine,” “(human OR participant),” “placebo,” and “self-administration,” again linked by the Boolean operator AND. This search generated a list of 20 records, and we excluded 11 studies classified as the wrong study type. We then assessed the 9 remaining reports, excluding 3 as they did not measure self-administration. Of the 25 putative medications originally identified in the clinical trials search, 5 pretreatments had also been examined in the human laboratory. In total, 6 human laboratory studies were included (Supplemental Fig. 1B).

Next, we examined the nonhuman primate (NHP) literature. To meet inclusion criteria, NHP studies had to incorporate some measure of methamphetamine self-administration and at least one of the candidate medications identified through the clinical trials. We searched for “methamphetamine,” “(monkey OR non-human primate OR NHP),” “self-administration,” and a list of all clinical trial medications (with pretreatments separated by the OR operator) using the Boolean operator AND. This search produced 54 records. Of these records, 48 were deemed the wrong study type and excluded. Of the 6 reports assessed for eligibility, 1 was excluded for not including self-administration, and 1 was excluded for using a treatment not identified in our original clinical trials search. Through this screening process, we found 4 medications that had been tested in clinical trials. As a result, we included 4 NHP studies in the present review (Supplemental Fig. 1C).

Finally, we determined which medications evaluated in the human laboratory and/or NHP laboratory had also been assessed in rodents. Consistent with prior research contexts, rodent studies met criteria if they had used one of the medications identified in the clinical trials as well as the human laboratory and/or NHP laboratory as a medication for methamphetamine self-administration. Studies that only examined the impact of pretreatments on acquisition (initiation), extinction, or reinstatement were excluded. Our search terms were “methamphetamine, “(rodent OR rat OR mouse OR mice),” “self-administration,” and a list of all clinical trial medications (each separated by OR) connected by the Boolean operator AND. This search yielded 85 records; 42 studies (such as reviews and papers involving nonrodent subjects) were immediately excluded. Forty-three reports were evaluated, and 16 were excluded for failing to measure medications’ effects on self-administration, whereas 20 were excluded for using a treatment not previously identified in other research contexts. In sum, we identified 5 medications across 7 rodent studies that had been tested in clinical trials as well as the human laboratory and/or NHP laboratory (Supplemental Fig. 1D). We used these 5 clinically tested medications that had also been assessed across human laboratory and preclinical contexts to analyze the translational methamphetamine medications development pipeline.

### C Treatment efficacy analysis strategy

When applicable, we highlighted the following 6 key study details: (1) pretreatment characteristics (eg, dose, dosing schedule, and route of administration); (2) quantification of methamphetamine use (eg, weekly percent positive score for urinalysis or number of methamphetamine lever presses emitted for self-administration); (3) methamphetamine dose and route of administration for self-administration; (4) study design (timelines, tasks, and schedules of reinforcement); and (5) sample size. This information appears in Table 1^74^, ^75^, ^76^, ^77^, ^78^, ^79^, ^80^, ^81^, ^82^, ^83^, ^84^, ^85^, ^86^, ^87^, ^88^, ^89^, ^90^, ^91^, ^92^, ^93^, ^94^, ^95^, ^96^, ^97^, ^98^, ^99^, ^100^, ^101^ and is further described in the Results and Discussion sections. In this manuscript, treatment effects were considered significant when *P* < .05. Based on the main outcomes identified above, individual studies were coded as either “+” (eg, they provided positive data for the medication as a putative treatment for MUD) or “−” (eg, they provided negative data for the medication as a putative treatment for MUD; Table 2). We also created Supplemental Table 1 akin to Table 2 for medications identified in clinical trials but not evaluated across all 3 research settings. The same scoring guidelines applied.

**Table 1:** Selected study designs

Research Context	Treatment Characteristics	Measure of Meth Use	MA Dose and ROA	Task and Schedules of Reinforcement	Sample Size	Reference
Aripiprazole						
Clinical trial	Target daily dose of 20 mg, oral, or placebo for 12 wk	MA urine drug test results	N/A	N/A	90	Coffin et al, 2013^74^
Clinical trial	Target daily dose of 5–10 mg (titrated up/reduced as needed; no ROA specified) or placebo for 8 wk	MA urine drug test results	N/A	N/A	37	Sulaiman et al, 2013^75^
Human laboratory	Administration of 0 and 15 mg, oral, over 16 sessions	Breakpoint	0, 4, 8, 16 mg, oral	PR SA (MA only)	7	Stoops et al, 2013^76^
Preclinical laboratory	Pretreatment with 0, 0.3, 1, 3, 10 mg/kg, s.c., 1 h before sessions	No. of infusions	0, 0.025, 0.05, 0.1, 0.2 mg/kg/inf., i.v.	FR5 and PR SA (MA only) after short (1 h) and long (6 h) access conditions	28	Wee et al, 2007^77^
Bupropion						
Clinical trial	Target daily dose of SR 300 mg, oral, or placebo for 12 wk	MA urine drug test results	N/A	N/A	151	Elkashef et al, 2008^78^
Clinical trial	Target daily dose of SR 300 mg, oral, or placebo for 12 wk	MA urine drug test results	N/A	N/A	73	Shoptaw et al, 2008^79^
Clinical trial	Target daily dose of XL 300 mg, oral, or placebo for 12 wk	MA urine drug test results	N/A	N/A	30	Das et al, 2010^80^
Clinical trial	Target daily dose of SR 300 mg, oral, or placebo for 12 wk	MA urine drug test results	N/A	N/A	204	Anderson et al, 2015^81^
Human laboratory	Target daily dose of 300 mg, oral*,* or placebo with 4 days of maintenance	Breakpoint	0, 10, 30 mg, i.n.	PR SA (MA only)	7	Stoops et al, 2015^82^
Preclinical laboratory	Dosing with 0, 10, 30, 60 mg/kg, i.p.*,* 5 min before session start and repeated treatment with 30 and 60 mg/kg, i.p.*,* for 3 and 5 days, respectively	No. of infusions	0.025, 0.05, 0.1 mg/kg/inf., i.v.	FR5 SA (MA and sucrose separately)	36 (27)	Reichel et al, 2009^83^
Preclinical laboratory	Administration of 0, 1, 3.2, 10 mg/kg, i.m.*,* 20 min before session start	MA intake (response rates)	0, 1, 3, 10, 30 *μ*g/kg/inf., i.v.	FR10 MA and food SA (alternating schedule – not concurrent)	8	Schindler et al, 2011^84^
Preclinical laboratory	Administration of 0, 0.32, 1.0, 1.8 mg/kg/h, i.v., for 7 days	Percent MA choice and no. of choices	0, 0.01, 0.032, 0.1, 0.32 mg/kg/inf., i.v.	Concurrent MA – food choice task	6	Banks and Blough, 2015^85^
Preclinical laboratory	Administration of 0, 10, 30, 60 mg/kg, i.p.*,* 10 min before session start	No. of active lever presses	0.05 mg/kg/inf., i.v.	FR5 SA (MA and sucrose separately)	17 (10)	Charntikov et al, 2018^86^
*D*-amphetamine						
Clinical trial	Target daily dose of SR 60 mg, oral, or placebo for 8 wk	MA urine drug test results	N/A	N/A	60	Galloway et al, 2011^87^
Human laboratory	Target daily dose of SR 40 mg, oral, or placebo after 7 days of maintenance	Breakpoint	0, 10, 20, 30, i.n.	PR SA (MA only)	8	Pike et al, 2014^88^
Preclinical laboratory	Administration of 0, 0.1, 0.32, 1.0 mg/kg, i.p.*,* before session start	No. of active lever presses per second	0, 0.01, 0.032, 0.10, 0.32 mg/kg/inf., i.v.	FR5 SA (MA only)	30 (24)	Hiranita et al, 2014^89^
Preclinical laboratory	Administration of 0, 0.01, 0.032, 0.1 mg/kg/h, i.v.*,* for 7 days	Percent MA choice and no. of choices	0, 0.01, 0.032, 0.1, 0.32 mg/kg/inj., i.v.	Concurrent MA – food choice	4	Schwienteck and Banks, 2015^90^
Modafinil						
Clinical trial	Target daily dose of 200 mg, oral, or placebo for 10 wk	MA urine drug test results	N/A	N/A	80	Shearer et al, 2009^91^
Clinical trial	Target daily dose of 400 mg, oral, or placebo for 12 wk	MA urine drug test results	N/A	N/A	71	Heinzerling et al, 2010^92^
Clinical trial	Target daily dose of 200 mg or 400 mg, oral*,* or placebo for 12 wk	MA urine drug test results	N/A	N/A	210	Anderson et al, 2012^93^
Human laboratory	Target daily dose of 200 mg, oral, or placebo for 3 days each	No. of MA choices and monetary value of MA	0, 3.0 mg, i.v.	MA-money choice task	13	De La Garza et al, 2010^94^
Preclinical laboratory	Pretreatment with 0, 30, 100, 300 mg/kg, i.p.*,* 90 min before session start	MA intake and no. of active lever presses	20 *μ*g/50 *μ*L, i.v.	FR1 SA (MA only)	12	Reichel and See, 2012^95^
Preclinical laboratory	Administration of 0 or 100 mg/kg, i.p., 1 h before session start	No. of MA infusions	0.05 mg/kg/inf., i.v.	FR1 SA (MA only) under short (1 h) or long (6 h) access conditions	29 (21)	Tunstall et al, 2018^96^
Naltrexone						
Clinical trial	Administration of XR 380 mg, i.m.*,* or placebo once every 4 wk over a 12-wk trial	MA urine drug test results	N/A	N/A	100	Coffin et al, 2018^97^
Human laboratory	Target daily dose of 50 mg, oral*,* or placebo for 4 days of maintenance	Breakpoint	0, 10, 30 mg, i.n.	PR SA (MA only)	7	Stoops et al, 2015^82^
Human laboratory	Target daily dose of 50 mg, oral*,* or placebo for 6–7 days of maintenance	No. of MA choices	0, 10, 30 mg, i.n.	PR SA (MA only)	8	Marks et al, 2016^98^
Human laboratory	Pretreatment with 0 or 50 mg, oral, 1 h before session start	Breakpoint	30 mg/70 kg, i.n.	MA-money choice task	14	Jones et al, 2022^99^
Preclinical laboratory	Continuous infusions (progressing from 0, 0.25, 1.0, 2.5, up to 10 *μ*g/kg/h, i.v.) for 4 wk	No. of MA infusions	4 *μ*g*/*kg/inf., i.v.	FR SA (MA only)	5	Harrigan and Downs, 1978^100^
Preclinical laboratory	Administration of 0, 20, 40 mg/kg, i.g.*,* 30 min before session start	No. of MA infusions and active pokes per 20 min	0, 0.00625, 0.0125, 0.025, 0.05, 0.1 mg/kg/inf., i.v.	FR2 SA (MA only)	7	Guo et al, 2019^101^

Study design details are depicted in this table when applicable, with special attention paid to pretreatment drug characteristics (dose, dosing schedule, and route of administration), measures of methamphetamine use, methamphetamine dose and route of administration, self-administration assay (task structure and schedules of reinforcement, and sample size). For clinical trial and human laboratory settings, treatment characteristics column lists each study’s target daily dose. Within the sample size column, 2 values are provided for preclinical studies that used between-subjects designs to test the effects of a drug on methamphetamine and sucrose self-administration separately (eg, behavioral selectivity). The first value denotes the combined sample size for both groups, and the value in parentheses denotes the number of subjects that self-administered methamphetamine alone. Preclinical rodent studies are italicized.

inf., infusion; MA, methamphetamine; N/A, not applicable; ROA, route of administration; SA, self-administration; SR, sustained release; XR, extended release.

**Table 2:** Published studies included in this review

Drug	Preclinical Studies	Human Laboratory Studies	Clinical Trials
Drugs that target monoamines:			
Aripiprazole	*(+)Wee et al, 2007*^77^	(+)Stoops et al, 2013^76^	(−)Coffin et al, 2013^74^
			(−)Sulaiman et al, 2013^75^
Bupropion	*(+)Reichel et al, 2009*^83^	(−)Stoops et al, 2015^82^	(−)Elkashef et al, 2008^78^
	(+)Schindler et al, 2011^84^		(−)Shoptaw et al, 2008^79^
	(−)Banks and Blough, 2015^85^		(−)Das et al, 2010^80^
	*(−)Charntikov et al, 2018*^86^		(−)Anderson et al, 2015^81^
*D*-amphetamine	*(−)Hiranita et al, 2014*^89^	(−)Pike et al, 2014^88^	(−)Galloway et al, 2011^87^
	(−)Schwienteck and Banks, 2015^90^		
Modafinil	*(+)Reichel and See, 2012*^95^	(−)De La Garza et al, 2010^94^	(−)Shearer et al, 2009^91^
	*(+)Tunstall et al, 2018*^96^		(−)Heinzerling et al, 2010^92^
			(−)Anderson et al, 2012^93^

Drugs that target other mechanisms:			
Naltrexone	(−)Harrigan and Downs, 1978^100^	(−)Stoops et al, 2015^82^	(−)Coffin et al, 2018^97^
	*(−)Guo et al, 2019*^101^	(+)Marks et al, 2016^98^	
		(−)Jones et al, 2022^99^	

Symbols indicate whether studies provided positive (+) or negative (−) results about the drug’s suitability as a putative treatment for MUD. Under “Preclinical Studies,” investigations with rodent subjects are italicized. Studies that resulted in nonselective changes in self-administration of appetitive commodities (eg, those whose effects were not specific to methamphetamine) were coded as negative results. Likewise, studies where drug treatments increased methamphetamine self-administration were scored as negative results.

We then calculated a composite therapeutic promise score for each treatment within each research domain. For these composite scores, we used the same criteria outlined in prior work.^46^ When >50% of the studies for a certain treatment-context were positive, groups were categorized as decreasing methamphetamine self-administration. When <50% of the studies for a given treatment-context were positive, groups were categorized as having no significant effect on methamphetamine self-administration. Treatment-context groups that reported an equal number (= 50%) of positive and negative outcomes were coded as “mixed” (Table 3). Only group-level effects were considered when assessing pretreatment efficacy. However, findings from papers that also performed subgroup analyses are considered in the Discussion.

**Table 3:** Net effects of treatments on meth consumption

Drug	Animal Laboratory (11)	Human Laboratory (6)	Clinical Trials (11)
Aripiprazole	⇓ (1/1)^77^	⇓ (1/1)^76^	- (0/2)^74^^,^^75^
Bupropion	⇔ (2/4)^83^, ^84^, ^85^, ^86^	- (0/1)^82^	- (0/4)^78^, ^79^, ^80^, ^81^
*d*-amphetamine	⇔(0/2)^89^^,^^90^	- (0/1)^88^	- (0/1)^87^
Modafinil	⇓ (2/2)^95^^,^^96^	- (0/1)^94^	- (0/3)^91^, ^92^, ^93^
Naltrexone	- (0/2)^100^^,^^101^	- (1/3)^82^^,^^98^^,^^99^	- (0/1)^97^

The values in parentheses show the number of studies reporting significant decreases in methamphetamine intake (numerator) out of all selected studies (denominator) for a given research context. Further, the “⇓” symbol indicates that the drug reduced methamphetamine taking (selectively, when applicable) in the majority of selected studies, the “-“ symbol shows that the drug did not alter methamphetamine use in the majority of selected studies, and the “⇔” symbol indicates that the drug had mixed effects on methamphetamine taking across selected studies. ^+^*d*-amphetamine treatment increased d-methamphetamine; self-administration (caused a leftward shift in methamphetamine dose-effect curve).

## III Results

### A Drugs targeting monoamines

#### 1 Aripiprazole

To characterize the effects of aripiprazole, a partial D_2_/5-HT_1A_ agonist and 5-HT_2A_ antagonist,^102^ on methamphetamine self-administration, laboratory rats were treated with aripiprazole (0.3–10 mg/kg, s.c.) prior to daily test sessions.^77^ One group of rats was trained under short-access (1-hour) conditions to produce a stable pattern of drug intake, whereas a second group of rats was trained under long-access (6-hour) conditions to produce a phenotype more characteristic of addiction.^103^^,^^104^ On both FR and PR schedules of reinforcement, aripiprazole shifted the methamphetamine dose-effect curve downward and to the right in both groups, but these effects were more pronounced in rats trained under long-access conditions. Control measures of responding (eg, inactive lever responding and food-maintained responding) were not collected, and thus the degree of selectivity for drug- versus nondrug-reinforced responding could not be determined.

Likewise, when participants in a human laboratory study received pretreatments of aripiprazole (0 or 15 mg, orally*)*, it attenuated methamphetamine breakpoints on a PR self-administration task (0–16 mg, orally).^76^ However, when 2 clinical trials^74^^,^^75^ administered aripiprazole orally at doses of 5 to 20 mg daily over 8 to 12 weeks, it did not change the proportion of methamphetamine positive urine samples to a greater degree than placebo.

#### 2 Bupropion

The effects of the norepinephrine-dopamine reuptake inhibitor bupropion^105^ on methamphetamine use were similarly evaluated. In rats, bupropion (10–60 mg/kg, i.p.) has been tested across 2 studies.^83^^,^^86^ Pretreatment administration began either immediately before the experimental session^82^ or for up to 5 days preceding the experimental session.^83^ In both studies, sucrose-maintained responding was tested in separate groups to determine the selectivity of bupropion to reduce drug- versus nondrug-reinforced responding, and both methamphetamine and sucrose were available on FR schedules of reinforcement. Bupropion dose-dependently decreased methamphetamine intake in both studies, but it only selectively decreased methamphetamine intake without decreasing sucrose intake in 1 study,^83^ and only at 1 dose (30 mg/kg). In both studies, 60 mg/kg bupropion decreased responding maintained by both methamphetamine and sucrose.

Two studies have examined the effects of bupropion on methamphetamine-maintained responding in rhesus monkeys. Similar to that reported in rats, a moderate dose of bupropion (3.2 mg/kg, i.m.) selectively decreased methamphetamine self-administration on an FR schedule without altering responding maintained by food.^84^ In a food versus methamphetamine choice procedure, continuous bupropion (0.32–1.8 mg/kg/h, i.v., across 7 days) decreased methamphetamine intake at the highest treatment dose, but decreased food intake by a comparable amount, with the authors concluding that bupropion failed to decrease methamphetamine versus food choice.^85^

One human laboratory study^82^ found that maintenance on bupropion (300 mg/day) did not change intranasal methamphetamine (0–30 mg) self-administration during a modified PR task. Bupropion has been tested in 4 separate clinical trials.^79^, ^80^, ^81^ In each of those 12-week trials, oral bupropion (300 mg target daily dose) failed to significantly change the proportion of methamphetamine positive urine samples to a greater degree than placebo.

#### 3 D-amphetamine

Four studies assessed the effects of *d*-amphetamine, which alters dopaminergic signaling by preventing reuptake and amplifying release,^106^ on methamphetamine consumption. In a single study conducted in rats, administration of *d*-amphetamine (0.1–1 mg/kg, i.p.) shifted the methamphetamine dose-effect curve to the left on an FR schedule of reinforcement, indicating an increase in the potency of methamphetamine to serve as a positive reinforcer.^89^ In a single study conducted in rhesus monkeys, continuous *d*-amphetamine pretreatment (0.01–0.1 mg/kg/h, i.v., for 7 days) altered methamphetamine versus food choice differentially across the 4 monkeys tested. In 2 monkeys, the highest dose of *d*-amphetamine produced a left-ward shift in the methamphetamine choice curve, indicating an increase in methamphetamine’s relative reinforcing efficacy compared with the alternative reinforcer, food. However, this dose of *d*-amphetamine eliminated methamphetamine choice in the other 2 monkeys, such that food was selected over all methamphetamine doses tested.^90^

When people who used stimulants were maintained on *d*-amphetamine, they self-administered methamphetamine doses to a similar degree as was observed during placebo maintenance.^88^ Receiving 60 mg of sustained-release oral *d*-amphetamine daily during an 8-week clinical trial^87^ also failed to significantly change the proportion of methamphetamine positive urine samples relative to placebo.

#### 4 Modafinil

The norepinephrine-dopamine reuptake inhibitor modafinil served as another candidate medication for MUD evaluated across clinical trials, human laboratory, and preclinical laboratory contexts.^107^ Modafinil (administered either as racemic or as its active R-modafinil isomer) has been tested in 2 studies in rats responding on an FR schedule of reinforcement.^95^^,^^96^ Administration of racemic modafinil (30–300 mg/kg, i.p.) 90 minutes before experimental sessions decreased methamphetamine intake only at the highest dose tested; however, only a single dose of methamphetamine was tested (0.02 mg bolus infusions), and no control measures of responding were collected.^95^ In a second study, administration of R-modafinil (100 mg/kg) 1 hour before experimental sessions decreased responding maintained by methamphetamine in rats responding under short-access but not long-access conditions.^96^ Similar to the previous study, only a single dose of methamphetamine was tested (0.05 mg/kg/infusion), and no control measures of responding were collected. The latter rodent study did assess methamphetamine doses slightly higher than the maximal range compared with the dose-effect curve described in the literature.^108^, ^109^, ^110^, ^111^, ^112^

In contrast, results from a human laboratory study suggested that modafinil did not alter methamphetamine use. Namely, when participants who were dependent on methamphetamine and not seeking treatment received randomized, once-daily treatments of oral modafinil or placebo for 3 days each (separated by a 4-day washout period), methamphetamine self-administration was similar across treatment conditions.^94^ Similarly, negative outcomes were observed with oral modafinil (200–400 mg daily) across 3 trials that lasted 10 to 12 weeks.^91^, ^92^, ^93^

### B Drugs with other targets: Naltrexone

Two preclinical studies have examined the effects of the mu opioid receptor antagonist naltrexone on methamphetamine self-administration.^100^^,^^101^^,^^113^ Naltrexone (20 and 40 mg/kg, i.g.) administered 30 minutes before the experimental sessions failed to alter the methamphetamine dose-effect curve as determined in rats responding on an FR schedule of reinforcement.^101^ Similarly, repeated naltrexone pretreatment (0.25–10 *μ*g/kg per hour, i.v., across 4 weeks) failed to alter intake of a single dose of methamphetamine (0.004 mg/kg/infusion) in rhesus monkeys responding on an FR schedule of reinforcement.^100^

Three studies have evaluated the effect of naltrexone on methamphetamine self-administration in the human laboratory.^82^^,^^98^^,^^99^ Compared with placebo, oral naltrexone (50 mg) administered before the session did not alter operant intranasal methamphetamine self-administration (30 mg/70 kg) during a drug-money choice task.^99^ However, studies that used slightly longer dosing schedules yielded mixed findings.^82^^,^^98^ For example, oral naltrexone (50 mg a day for 6–7 days) reduced intranasal methamphetamine PR self-administration at lower doses (10 mg) but not higher doses (30 mg) relative to placebo.^98^ In contrast, 4 days of oral naltrexone (50 mg) maintenance did not affect intranasal methamphetamine self-administration (0–30 mg) on a PR task compared with placebo.^82^ Injected, extended-release naltrexone (380 mg administered at 4-week intervals) did not produce statistically different changes in methamphetamine positive urines relative to matched placebo in one 12-week clinical trial.^97^

## IV Discussion

In this review, we initially identified 25 candidate medications tested for MUD across 36 clinical trial reports; however, only 5 of these putative treatments had also been assessed in the human and animal laboratories. These drugs belonged to one of the following categories: drugs targeting monoamines and drugs with other targets (naltrexone).

### A Summary: Drugs targeting monoamines

Findings regarding the suitability of drugs involving monoamines as candidate medications for MUD varied between research contexts. Animal laboratory studies showed mixed effects, whereas all but 1 human laboratory study^76^ and all clinical trials showed no significant effect of treatment on methamphetamine-taking behaviors (Table 3). Differences in dosing regimens likely contributed to these discordant outcomes (see Table 1). For example, short-term aripiprazole pretreatment attenuated methamphetamine self-administration in the rodent^77^ and human laboratories,^76^ whereas repeated aripiprazole pretreatment (spanning 8–12 weeks) did not reduce methamphetamine use relative to placebo in clinical trials.^74^^,^^75^

Further, many animal laboratory studies failed to include measures of behavioral selectivity. When rats and NHPs received bupropion, it significantly reduced methamphetamine self-administration; however, these effects were only selective in 2 of the 4 studies evaluated,^83^^,^^84^ suggesting mixed effects (Table 3). Interestingly, the 2 rodent studies used overlapping bupropion pretreatment and methamphetamine dose ranges; in 1 study,^83^ bupropion (30 mg/kg) selectively attenuated methamphetamine self-administration (0.05 mg/kg/infusion) in male rats, whereas the same bupropion dose failed to selectively decrease self-administration (also at 0.05 mg/kg/infusion) in another study^86^ with female rats. These different outcomes highlight a possible sex difference in treatment efficacy. It is well established that drug-taking behavior varies in phase with the estrous cycle.^114^, ^115^, ^116^ Under a PR schedule of reinforcement, cocaine breakpoints decrease during estrus relative to other estrous cycle phases.^114^^,^^116^ Of note, estrous cycle phase does not impact responding for nondrug reinforcers, such as sugar, in female rats.^115^ Sex differences may impact drug intake and, by consequence, medication efficacy, contributing to these discrepant preclinical findings. In contrast, bupropion did not change methamphetamine use in a human laboratory study^82^ or clinical trials.^78^, ^79^, ^80^, ^81^ Similar trends emerged for modafinil, which reduced methamphetamine self-administration in rats,^95^^,^^96^ but neither study examined if these effects were specific to methamphetamine-taking behaviors. In contrast, modafinil failed to alter methamphetamine use in human laboratory^94^ or clinical trial contexts.^91^, ^92^, ^93^ Finally, *d*-amphetamine actually increased methamphetamine’s reinforcing effects in rats,^89^ and it had mixed effects on methamphetamine-food choice among NHPs^90^ (see Table 3). It did not affect methamphetamine use in human laboratory^88^ or clinical trial^87^ contexts.

Although these drugs did not alter methamphetamine use in clinical trials at the group level, some medications altered use at the subgroup level. Importantly, the FDA has acknowledged that the heterogeneity among people with stimulant use disorders such as MUD complicates the medications development pipeline; the efficacy of candidate treatments may vary for specific subpopulations based on factors such as preferred routes of administration or motivations for methamphetamine use.^50^ Consistent with this guidance, select clinical trials^78^^,^^79^^,^^81^^,^^92^ performed subgroup analyses examining the effects of candidate medications for people who reported lower baseline levels of methamphetamine use. Studies reported mixed findings. For example, bupropion significantly reduced methamphetamine use^79^ and increased methamphetamine abstinence among male participants^78^ relative to placebo. In contrast, methamphetamine use among individuals with lower baseline consumption did not change after receiving bupropion^81^ or modafinil^92^ relative to placebo. Limited data suggested that bupropion may benefit people with low baseline levels of methamphetamine use, but modafinil did not show promise in subgroup analyses.

Altogether, the animal laboratory findings showed mixed support for the advancement of drugs involving monoamines, but they contained crucial methodological limitations (discussed below in greater detail) that impacted concordance and have important implications for future design of preclinical screening for MUD pharmacotherapies. When considered in combination with the human laboratory and clinical trial data, the cumulative findings provided negative results about drugs involving monoamines as putative treatments for MUD at the group level, but further research targeting subgroup will acknowledge the heterogeneity characterizing SUDs and refine medications development.

### B Summary: Drugs with other targets

The preclinical, human laboratory, and clinical trial data showed consistent effects of naltrexone on methamphetamine use (Table 3). All but one of these studies^98^ showed no effect of naltrexone pretreatment on methamphetamine self-administration across research contexts. Discrepant effects of naltrexone treatment in the human laboratory may be attributed to different dosing regimens (see Table 1). Namely, twice-daily dosing (50 mg total, orally) for 6 to 7 days of maintenance attenuated methamphetamine self-administration,^98^ whereas once-daily dosing (50 mg, orally) with 4 days of maintenance was not effective.^82^ Together, these animal laboratory, human laboratory, and clinical trial findings showed consistently negative results for the suitability of naltrexone as a putative pharmacotherapy for MUD.

### C Concordance

Prior reviews have highlighted the importance of evaluating medications development through a translational pipeline that helps bridge animal studies, human laboratory studies, and clinical trials.^46^^,^^117^, ^118^, ^119^ Within this pipeline, the primary goal of laboratory research is to identify promising candidate medications and dosing regimens to advance to clinical trials. Such a pipeline is vital for SUDs without FDA-approved treatments (such as MUD). Inherent to the translational medications development pipeline are 2 assumptions: that (1) good concordance exists between the animal laboratory, human laboratory, and clinical trials and (2) laboratory data provide a high degree of predictive validity regarding clinical trial outcomes.

We preface our discussion of concordance in the present review with 3 important caveats. First, we acknowledge the small number of studies that met criteria for this review. Each research domain contained 1 to 4 published studies per medication, which limited interpretations. For example, only 1 clinical trial^97^ evaluated naltrexone for MUD. None of the candidate medications had more than 1 study in all domains. The second pertains to predictive validity; importantly, as most selected studies provided negative evidence for the suitability of the study drugs for advancement as pharmacotherapies for MUD, it limits our ability to draw conclusions about positive predictive validity. Instead, discussing concordance highlights negative predictive validity (ie, if a drug fails to alter methamphetamine self-administration in the laboratory, it failed in clinical trials). Finally, some of the clinical trials in this review preceded laboratory studies, hindering our ability to assess predictive validity. In this review, analyzing validity often involves postdictive validity, not predictive validity.

#### 1 Preclinical—human laboratory concordance

Two drugs showed similar effects between animal and human laboratories. Aripiprazole decreased methamphetamine self-administration, whereas naltrexone did not significantly alter methamphetamine self-administration in most selected studies. However, the remaining 3 drugs (bupropion, *d*-amphetamine, and modafinil) showed discordant results between the preclinical and human laboratory domains. Notably, bupropion and *d*-amphetamine had mixed effects on preclinical methamphetamine self-administration, and modafinil attenuated preclinical self-administration; none of these candidate treatments altered human laboratory self-administration.

#### 2 Preclinical—clinical trial concordance

Similarly, candidate medications’ effects often differed between the animal laboratory and clinical trials. Two drugs (aripiprazole and modafinil) reduced methamphetamine self-administration in the animal laboratory without changing methamphetamine use relative to placebo in clinical trials, whereas 2 other drugs (bupropion and *d*-amphetamine) had mixed effects on methamphetamine self-administration in the animal laboratory without changing methamphetamine use relative to placebo in clinical trials. Naltrexone had no effect on methamphetamine use in either research domain.

#### 3 Human laboratory—clinical trial concordance

Results from the human laboratory and clinical trials showed a high degree of concordance, as 4 putative medications (bupropion, *d*-amphetamine, modafinil, and naltrexone) did not significantly change methamphetamine use in either domain. Aripiprazole alone attenuated methamphetamine self-administration in the human laboratory but not in clinical trials when administered repeatedly over several weeks.

#### 4 Summary

Overall, drug pretreatments were more likely to alter methamphetamine use in preclinical studies relative to the other research contexts, suggesting preclinical models could have lower ability to screen that medications will be effective in treating MUD. None of the 5 medications evaluated in this review showed consistent net reductions in methamphetamine use across preclinical, human laboratory, and clinical trial contexts. However, the effects of pretreatments on methamphetamine self-administration varied in the animal laboratory; methodological differences likely contributed to these discrepant findings, and they are discussed below. In the preclinical laboratory, 2 pretreatments (aripiprazole and modafinil) attenuated methamphetamine use, 2 medications (bupropion and *d*-amphetamine) had mixed effects on methamphetamine use, and 1 medication (naltrexone) showed no effect on methamphetamine use. In contrast, only 1 putative medication (aripiprazole) reduced methamphetamine self-administration in the human laboratory, whereas the other 4 did not change intake. Similarly, none of the candidate medications significantly reduced methamphetamine intake at the group level relative to placebo in any of the 11 identified clinical trials.

This analysis of concordance should be interpreted with caution. We reiterate the limitations of evaluating the translational methamphetamine medications development pipeline in the present review. Namely, the low number of studies across research domains for each drug makes it difficult to meaningfully assess concordance. Further, the preponderance of negative results, combined with the lack of evidence-based progression from the laboratory to clinical trials, only allowed us to demonstrate negative postdictive validity.

### D Overview of nonqualifying studies: Additional therapeutic targets

Only 20% of the medications identified from clinical trials for MUD had been evaluated across all research settings (see Fig. 1). The 5 medications discussed in the present review represented a subset of potential therapeutic targets for MUD. In addition to drugs that targeted monoamines and opioid receptors, our original clinical trials search included putative mediations that targeted GABAergic and glutamatergic mechanisms (such as gabapentin^120^ and riluzole^121^), along with other mechanisms, both alone and combined (eg, N-acetylcysteine – naltrexone^122^; please refer to Supplemental Table 1 for an exhaustive list).

Although none of the clinical trials included in the present review supported the advancement of the 5 selected candidate compounds for MUD medications development, data from some of the clinical trials in Supplemental Table 1 provided evidence to support further exploration of the efficacy of these putative pharmacotherapies. Notably, 3 drugs that targeted GABA-glutamate systems (riluzole,^121^ topiramate,^123^^,^^124^ and valproate^125^) showed consistently positive results for suitability. One drug that targeted monoamines showed positive results (mirtazapine^126^^,^^127^), whereas another (methylphenidate^128^^,^^129^) showed mixed support for advancement. Further, buprenorphine showed consistently positive results.^130^ Naltrexone-bupropion combined did not alter methamphetamine self-administration in the human laboratory^82^ but did reduce methamphetamine use in 1 clinical trial.^131^ This discrepancy may be because of lack of statistical power in the human laboratory study given that reductions in drug taking in both studies were strikingly similar (ie, 14% in the human laboratory study and 11% in the clinical trial).

### E Methodological considerations

It is likely that methodological issues in the extant MUD literature contributed to inconsistencies across levels of analysis, as well as to the lack of positive signals (ie, methamphetamine abstinence) observed in the reviewed studies. These methodological weaknesses and potential limitations, along with some recommendations for best practices are discussed below.

#### 1 Behavioral selectivity

Others^46^^,^^132^ have underscored the value of assessing behavioral selectivity in the medications development pipeline. Of the 11 preclinical studies reviewed, 8 studies reported a decrease in methamphetamine intake under at least 1 condition; however, several important caveats must be noted. Under conditions in which a putative medication reduced methamphetamine intake, it also often reduced food-reinforced responding at a similar or slightly higher (ie, ≤0.5 log unit) dose than that required to decrease methamphetamine-reinforced responding. Consequently, decreases in methamphetamine intake could be secondary to general decreases in motoric activity, or they may signal nonspecific reductions in the reinforcing effectiveness of appetitive commodities. As some of the selected studies in the present review did not include these measures, reductions in drug intake may have been misattributed to some therapeutic potential of the candidate medication when, in reality, they reflected nonselective drug effects on responding. It is of paramount importance to select assays that measure behavioral selectivity to maximize the utility of preclinical data when determining if putative medications warrant further investment.

#### 2 Dependent versus nondependent models

The preclinical rodent studies primarily relied on once-daily short access (1–2 hour) self-administration sessions, commonly regarded as a *nondependent* model of drug use; however, 2^77^^,^^96^ also included long-access conditions, a common proxy for methamphetamine dependence.^66^^,^^103^^,^^104^^,^^133^ In contrast, 4 of the 6 human laboratory studies, along with all clinical trials included in this review, recruited participants who met Diagnostic and Statistical Manual of Mental Disorders criteria for methamphetamine dependence/abuse (note: many of these studies were conducted before the Diagnostic and Statistical Manual of Mental Disorders-5 changed this terminology to MUD). Prior work^134^ showed that withdrawn conditions increased food-heroin choice in monkeys relative to nonwithdrawn conditions; in other words, using dependent and nondependent models may alter drug intake. However, as others have described in detail, opioid and stimulant dependence have unique neurobiological and behavioral features^135^; we must acknowledge that opioid dependence may exert greater influence over drug choice compared with stimulant dependence. Notably, a parallel cocaine study evaluated food-cocaine choice in monkeys before, during, and after periods of extended access (daily 21-hour long supplemental sessions of cocaine self-administration). Relative to baseline, behavior allocation between food and cocaine did not differ significantly during extended access conditions or during withdrawal from extended access conditions; in other words, cocaine withdrawal did not amplify cocaine’s reinforcing efficacy or alter levels of cocaine consumption. These data further corroborate the assertion that withdrawal may have unique effects on opioid and stimulant choice.^136^ In our review, the use of different models likely contributed to the poor concordance between the animal laboratory and other research domains. Further, dependent models improve face validity, enhancing the translational appeal of animal laboratory models for SUD medications development.

#### 3 Methamphetamine dose selection

Three of the preclinical studies reporting a decrease in methamphetamine intake only tested a single dose of methamphetamine on an FR schedule of reinforcement. Methamphetamine-reinforced responding on FR schedules is characterized by an inverted-U-shaped dose-effect curve, with both an ascending and descending limb. Decreases in responding at any one dose could reflect a downward shift in the dose-effect curve (a decrease in reinforcer efficacy), a rightward shift in the dose-effect curve (a decrease in reinforcer potency), or a leftward shift in the dose-effect curve (an increase in reinforcer potency). The latter possibility would be contraindicated for a potential medication. As described above, *d*-amphetamine shifted the methamphetamine dose-effect curve to the left in rats responding on an FR schedule,^89^ indicating an increase in reinforcer potency. This effect would have been missed and may have been misinterpreted as a decrease in reinforcer efficacy if only a single dose had been tested. Robust preclinical medications development protocols test full dose-effect functions whenever feasible.

#### 4 Treatment dosing regimen

The duration of drug pretreatment often varies between laboratory and clinical trial contexts. In the present review, all but one^100^ of the selected preclinical and human laboratory studies relied on short-term (acute) dosing regimens (eg, pretreatment prior to behavioral sessions with minimal-to-no maintenance), whereas all clinical trials used repeated (chronic) dosing or depot formulations over the course of 8 to 12 weeks. It is well established that the acute and chronic effects of drugs may vary; further, animal studies that use chronic dosing are uniquely positioned to identify signs of tolerance to putative medications’ therapeutic effects or sensitization to harmful effects.^46^ Discrepant dosing regimens between levels of analysis may have contributed to the mixed concordance between research domains in our study. Although we recognize that preclinical research designs are informed by constraints (such as catheter patency), selecting chronic dosing procedures enhances translation.

#### 5 Study retention and compliance

As others^46^ have described, another important difference between research domains is that putative medications are typically delivered noncontingently in the animal laboratory, whereas they are taken contingently in clinical trials. The former approach guarantees that preclinical subjects complete the full dosing regimen, but the latter is often associated with poor retention and medication adherence. Within our identified studies, clinical trials reported no between-group differences (eg, treatment vs placebo) for retention or compliance (when available); however, both values varied between studies. For people in study treatment arms, retention rates ranged from approximately 29%^91^ to 91%.^97^ Likewise, adherence varied from approximately 47%^81^ to 97%.^97^ It is important to note that definitions of retention and adherence also differed among studies. Nonetheless, both metrics are associated with therapeutic success.^137^ It is worth noting that the clinical trials often incorporated nonpharmacological interventions, such as group psychotherapy, whereas preclinical and human laboratory studies did not include this component. Behavioral interventions may improve retention and medication adherence^91^; identifying and using effective strategies to maximize these outcomes are of paramount importance.^138^

#### 6 Polydrug use and comorbid disorders

In the present review, we focused on putative medications evaluated to treat MUD alone, excluding clinical trials that recruited people who reported comorbid SUDs (other than alcohol, nicotine, and cannabis) or psychiatric disorders. Importantly, among the 151 clinical trial records identified, we only removed 7 studies based on reporting comorbid disorders (<5%). We acknowledge that polysubstance use is common^139^; in fact, people who use opioids are increasingly likely to also use methamphetamine,^140^ which complicates treatment strategies.^141^ However, as no FDA-approved pharmacotherapy for MUD exists and heterogeneity characterizes individual stimulant disorders,^50^ we elected to focus this review on MUD alone.

### F Future directions and recommendations

#### 1 Increasing investment in methamphetamine medications development

Overall, our findings suggest that the relative investment in MUD pharmacotherapies is very limited compared with other stimulant use disorders (eg, more than 60 medications had been tested in cocaine clinical trials by 2016)^46^ and other SUDs.^42^^,^^44^^,^^45^ No pharmacotherapies have received FDA approval for the treatment of stimulant use disorders, including MUD. Moreover, little guidance exists regarding MUD medications development, and therapeutic targets of interest remain unclear. Instead, medications development for other SUDs has received priority. For example, to quickly identify and develop new medications to combat the opioid crisis, the National Institute on Drug Abuse Division of Therapeutics and Medical Consequences published a list of their “ten most wanted” pharmacological targets. These targets were selected because they had the highest likelihood of receiving FDA approval for the treatment of opioid use disorder in the near term.^142^

Undeniably, opioid-related mortality is a major public health crisis; however, other-substance mortality has risen considerably and also warrants attention. Namely, noncocaine psychostimulant-related overdose deaths increased by 180% between 2015 and 2019.^2^ Further, between 1999 and 2021, methamphetamine-related mortality increased 50-fold; heroin/fentanyl coinvolvement also surged (reaching ∼62% by 2021).^3^ To facilitate greater investment in stimulant medications development, we encourage development of a list of “most wanted” therapeutic targets for rapid MUD medications development akin to what has been previously published for opioid use disorder.^142^ Studies inspired by this guidance could adapt pre-existing preclinical medications development testing algorithms^132^ to enhance translational appeal and subsequently use those paradigms to inform the translational medications development pipeline,^46^ prioritizing specific medications (and therapeutic targets) for further investment at each level of analysis.

#### 2 Nonabstinence outcomes

The primary outcome measure of clinical trials for SUD is generally drug-free urine screens—a measure that reflects complete abstinence from the drug. Complete abstinence is not a realistic measure of treatment efficacy in preclinical studies. Consequently, other outcome variables must be prioritized for preclinical-to-clinical translation.

It is notable that clinical trials do not directly address the diagnostic criteria of SUD as a primary outcome measure. Of the 11 diagnostic criteria for SUD, 2 are pharmacological criteria dealing directly with drug use (ie, tolerance and withdrawal), 8 are behavioral criteria dealing directly with drug use (eg, substance use in situations in which it is physically hazardous), and 1 is a cognitive criterion (ie, craving) that cannot be assessed directly in nonhuman animals. If craving is exempted, then sustained abstinence as indicated by drug-free urine screens means that 10 of the 11 diagnostic criteria for SUD must be eliminated to show a positive therapeutic response—an efficacy threshold that is markedly higher than that used for medications development for other neuropsychiatric disorders.^143^, ^144^, ^145^ For example, achieving at least a 50% reduction in symptom score severity compared with baseline on the 17-item Hamilton Depression Rating Scale and the Hamilton Anxiety Rating Scale is a well recognized endpoint for medications development for depression^144^ and generalized anxiety disorder, respectively.^143^ Similarly, partial score reductions in validated scales for attention deficit hyperactivity disorder (such as the ADHD-RS-5) serve as an important outcome when evaluating candidate medications’ suitability.^145^

A wide range of preclinical models of substance use is now available, and these models can either directly or indirectly evaluate all diagnostic criteria for SUD (see prior reviews^133^). A converging operations approach in which potential medications are tested in each of these various models would indicate which drugs may be most useful for targeting individual features of SUD, an approach that would be useful for a personalized medicine strategy. We emphasize that this goal cannot be achieved if complete abstinence is the criterion for treatment efficacy.

The FDA recently proposed that partial reductions in stimulant use may be a meaningful alternative endpoint to complete abstinence.^50^ How these novel endpoints are defined remains to be elucidated; this guidance only specifies that endpoints must demonstrate clinical benefit. A growing body of evidence supports the advancement of nonabstinence endpoints for stimulant use disorder medications development. The literature suggests that individuals who meet or exceed a threshold of 75% cocaine-negative urine tests during treatment are more likely to remain in treatment, report longer periods of continuous abstinence, and achieve better psychosocial outcomes relative to peers who do not meet this threshold.^146^ A 12-week randomized controlled trial using contingency management to treat moderate to severe cocaine use disorder similarly found that reductions in use (eg, changes in the weekly percentage of cocaine-positive urine screens over the course of the study) were associated with improved cardiovascular health,^147^ immune function,^148^ and quality of life, including psychosocial functioning and sleep.^149^ Another clinical trial is underway to characterize the effects of reductions in methamphetamine use on health and psychosocial function (NCT07226596).

More broadly, the FDA has formally recognized nonabstinence outcomes (eg, at least 2 World Health Organization risk drinking level reductions) as a primary endpoint for alcohol clinical trials.^150^^,^^151^ It is well established that World Health Organization risk drinking level reductions serve as reliable surrogate markers of health and psychosocial function; further, people perceive nonabstinence outcomes as more attainable than traditional endpoints, such as total abstinence or no heavy drinking days.^151^ Other regulatory bodies, such as the European Medicines Agency, have also recognized nonabstinence endpoints.^151^^,^^152^ Adopting the 2-level World Health Organization risk drinking level endpoint resulted in the approval of a fourth medication for alcohol use disorder, nalmefene, in Europe^153^^,^^154^ and Japan.^155^^,^^156^

A reduction in drug use without complete abstinence is associated with positive outcomes in human populations and are more amenable to evaluation in preclinical models. Adopting regulatory guidelines regarding nonabstinence endpoints in clinical trials has advanced medications development for the treatment of SUD, particularly alcohol use disorder. Additional work is warranted to identify and validate meaningful nonabstinence endpoints in clinical trials for stimulant use disorder. Recognizing harm reduction-informed, primary nonabstinence endpoints may help enhance investment in medications development for MUD by facilitating FDA approval of new and effective pharmacotherapies.

#### 3 Reiterating the value of the translational pipeline

Although the preclinical to clinical pipeline has not been successful at identifying potential medications for MUD, the pipeline has been successful at identifying nonpharmacological interventions for MUD, even when drug-free urine screens were used as the primary outcome measure in clinical trials. For instance, forced-choice procedures require a subject to abstain from drug self-administration to obtain a nondrug reinforcer (eg, sucrose, access to a social partner), thus modeling contingency management interventions that require an individual of abstain from drug use (as verified by drug-free urine screens) to obtain nondrug reinforcement (eg, money). When evaluating the effects of candidate medications on substance use, medications development should incorporate choice models in conjunction with other models, such as progressive ratio, punishment, reacquisition, and extended access models, using a converging operations approach. Choice tasks provide a unique measure compared with other procedures (eg, FR1); namely, they quantify the reallocation of time and effort devoted to drugs relative to other available commodities, mirroring reductions in social, occupational, or recreational activities characteristic of SUDs. Future work should also consider incorporating more complex schedules of reinforcement, such as Differential Reinforcement of Other Behavior or Differential Reinforcement of Lower Rates of Behavior, to reflect the contingencies that impact behavior during contingency management treatment. Preclinical studies,^90^ human laboratory studies,^157^ and clinical trials^158^ report a high degree of concordance in the ability of choice interventions to significantly decrease methamphetamine intake, confirming that positive predictive validity can be obtained in models of MUD, and demonstrating successful forward and backward translation between preclinical studies and clinical trials.

## Conflict of interest

William W. Stoops has consulted for Anebulo and Myosin on clinical trial design. All other authors declare no conflicts of interest.
