# Supplementary material for: Translational medications development for methamphetamine use disorder: A systematic review of preclinical, human laboratory, and clinical research
Source: Pharmacol Rev. 2026 May 27;78(4):100145. doi: 10.1016/j.pharmr.2026.100145 (PMC13632057; doi:10.1016/j.pharmr.2026.100145)
Supplement: Supplementary file 1 [file mmc1.docx]

**Supplemental Information**

**Supplemental Figure 1A. PRISMA Chart for Clinical Trials**

**Identification of studies via databases and registers**

Records removed *before screening*:

Duplicate records removed (n = 0)

Records identified from:

Databases (n = 151)

**Identification**

Records screened

(n = 151)

Records excluded

(n = 79)

Reports sought for retrieval

(n = 72)

Reports not retrieved

(n = 0)

**Screening**

Reports assessed for eligibility

(n = 72)

Reports excluded:

Comorbid disorder(s) (n = 7)

Non-dependent (n = 1)

No urinalysis (n = 28)

Wrong treatment (n = 25)

Studies included in review

(n = 11)

**Included**

**Supplemental Figure 1B. PRISMA Chart for Human Laboratory Studies**

**Identification of studies via databases and registers**

Records removed *before screening*:

Duplicate records removed (n = 0)

Records identified from:

Databases (n = 20)

**Identification**

Records screened

(n = 20)

Records excluded

(n = 11)

Reports sought for retrieval

(n = 9)

Reports not retrieved

(n = 0)

**Screening**

Reports assessed for eligibility

(n = 9)

Reports excluded:

No self-administration (n = 3)

Wrong treatment (n = 0)

Studies included in review

(n = 6)

**Included**

**Supplemental Figure 1C. PRISMA Chart for the Non-Human Primate Laboratory**

**Identification of studies via databases and registers**

Records removed *before screening*:

Duplicate records removed (n = 0)

Records identified from:

Databases (n = 54)

**Identification**

Records screened

(n = 54)

Records excluded

(n = 48)

Reports sought for retrieval

(n = 6)

Reports not retrieved

(n = 0)

**Screening**

Reports assessed for eligibility

(n = 6)

Reports excluded:

No self-administration (n = 1)

Wrong treatment (n = 1)

Studies included in review

(n = 4)

**Included**

**Supplemental Figure 1D. PRISMA Chart for the Rodent Laboratory**

**Identification of studies via databases and registers**

Records removed *before screening*:

Duplicate records removed (n = 0)

Records identified from:

Databases (n = 85)

**Identification**

Records screened

(n = 85)

Records excluded

(n = 42)

Reports sought for retrieval

(n = 43)

Reports not retrieved

(n = 0)

**Screening**

Reports assessed for eligibility

(n = 43)

Reports excluded:

No self-administration (n = 16)

Wrong treatment (n = 20)

Studies included in review

(n = 7)

**Included**

**Supplemental Table 1.** Published Methamphetamine Self-administration Studies with Pretreatments Not Evaluated across Research Contexts

| **Drug** | **Preclinical Studies** | **Human Laboratory Studies** | **Clinical Trials** |
| --- | --- | --- | --- |
| **Drugs that Target GABA-Glutamate Systems:** | | | |
| **Gabapentin** |  |  | (-)Heinzerling et al., 2006 |
| **N-acetylcysteine** | *(-)Charntikov et al., 2018* |  | (-)Mousavi et al., 2015 |
| **Riluzole** |  |  | (+)Farahzadi et al., 2019 |
| **Topiramate** |  |  | (+)Elkashef et al., 2012 |
|  |  |  | (+)Rezaei et al., 2016 |
| **Valproate** |  |  | (+)Kheirabadi et al., 2016 |
| **Drugs that Target Monoamines:** | | | |
| **Cabergoline** |  |  | (-)Alipour et al., 2021 |
| **Lisdexamfetamine** |  |  | (-)Ezard et al., 2025 |
| **Methylphenidate** | (-)Schindler et al., 2011 |  | (-)Ling et al., 2014 |
|  | (-)Schwienteck et al., 2015 |  | (+)Rezaei et al., 2015 |
| **Mirtazapine** |  |  | (+)Colfax et al., 2011 |
|  |  |  | (+)Coffin et al., 2020 |
| **Ondansetron** |  |  | (-)Johnson et al., 2008 |
| **Paroxetine** |  |  | (-)Piasecki et al., 2002 |
| **Sertraline** |  |  | (-)Shoptaw et al., 2006 |
| **Drugs that Target other Mechanisms:** | | | |
| **Buprenorphine** | *(+)Wang et al., 2022* |  | (+)Salehi et al., 2015 |
|  |  |  | (+) Kheirabadi et al., 2021 |
| **Cytidine-5'-diphosphate choline (CDP-choline)** |  |  | (-)Yoon et al., 2010 |
|  |  |  | (-)Jeong et al., 2021 |
| **Ibudilast** | *(+)Snider et al., 2013* |  | (-)Heinzerling et al., 2020 |
|  | *(+)Wang et al., 2025* |  |  |
| **Oxytocin** | *(+)Carson et al., 2010* |  |  |
|  | *(+)Cox et al., 2013* |  | (-)Stauffer et al., 2020 |
|  | *(+)Cox et al., 2017* |  |  |
|  | *(+)Westenbroek et al., 2017* |  |  |
|  | *(+)Everett et al., 2020* |  |  |
|  | *(+)Everett et al., 2021* |  |  |
| **Varenicline** | *(-)Pittenger et al., 2016* |  | (-)Briones et al., 2018 |
|  | *(-)Pittenger et al., 2017* |  |  |
|  | *(-)Kangiser et al., 2018* |  |  |
| **Miscellaneous Drug Combinations:** | | | |
| **Flumazenil, Gabapentin & Hydroxyzine** |  |  | (-)Ling et al., 2012 |
| **N-acetylcysteine - naltrexone** |  |  | (-)Grant et al., 2010 |
| **Naltrexone – bupropion** |  | (-)Stoops et al., 2015 | (+)Trivedi et al., 2021 |

*Note:* Symbols show whether studies provided positive (+) or negative (-) results about the drug’s suitability as a candidate pharmacotherapy for MUD. Studies involving rodent subjects are italicized. Studies that resulted in non-specific changes in self-administration (such as those whose effects were not specific to methamphetamine) were coded as negative results. Similarly, studies where drug treatments increased methamphetamine self-administration were scored as negative results.

**Supplemental References**

Alipour M, Jafarian M, Rastgoo R, et al. Cabergoline in Treatment of Methamphetamine-Dependent Patients and Its Effect on Serum Level of Glial Cell-Derived Neurotrophic Factor: A Randomized, Double-Blind, Placebo-Controlled Clinical Trial. *Eur Addict Res*. 2021;27(6):457-468. doi:10.1159/000515398

Briones M, Shoptaw S, Cook R, et al. Varenicline treatment for methamphetamine dependence: A randomized, double-blind phase II clinical trial. *Drug Alcohol Depend*. 2018;189:30-36. doi:10.1016/j.drugalcdep.2018.04.023

Carson DS, Cornish JL, Guastella AJ, Hunt GE, McGregor IS. Oxytocin decreases methamphetamine self-administration, methamphetamine hyperactivity, and relapse to methamphetamine-seeking behaviour in rats. *Neuropharmacology*. 2010;58(1):38-43. doi:10.1016/j.neuropharm.2009.06.018

Charntikov S, Pittenger ST, Pudiak CM, Bevins RA. The effect of N-acetylcysteine or bupropion on methamphetamine self-administration and methamphetamine-triggered reinstatement of female rats. *Neuropharmacology*. 2018;135:487-495. doi:10.1016/j.neuropharm.2018.03.021

Coffin PO, Santos GM, Hern J, et al. Effects of Mirtazapine for Methamphetamine Use Disorder Among Cisgender Men and Transgender Women Who Have Sex With Men: A Placebo-Controlled Randomized Clinical Trial. *JAMA Psychiatry*. 2020;77(3):246-255. doi:10.1001/jamapsychiatry.2019.3655

Colfax GN, Santos GM, Das M, et al. Mirtazapine to reduce methamphetamine use: a randomized controlled trial. *Arch Gen Psychiatry*. 2011;68(11):1168-1175. doi:10.1001/archgenpsychiatry.2011.124

Cox BM, Bentzley BS, Regen-Tuero H, See RE, Reichel CM, Aston-Jones G. Oxytocin Acts in Nucleus Accumbens to Attenuate Methamphetamine Seeking and Demand. *Biol Psychiatry*. 2017;81(11):949-958. doi:10.1016/j.biopsych.2016.11.011

Cox BM, Young AB, See RE, Reichel CM. Sex differences in methamphetamine seeking in rats: impact of oxytocin. *Psychoneuroendocrinology*. 2013;38(10):2343-2353. doi:10.1016/j.psyneuen.2013.05.005

Elkashef A, Kahn R, Yu E, et al. Topiramate for the treatment of methamphetamine addiction: a multi-center placebo-controlled trial. *Addiction*. 2012;107(7):1297-1306. doi:10.1111/j.1360-0443.2011.03771.x

Everett NA, Carey HA, Cornish JL, Baracz SJ. Sign tracking predicts cue-induced but not drug-primed reinstatement to methamphetamine seeking in rats: Effects of oxytocin treatment. *J Psychopharmacol*. 2020;34(11):1271-1279. doi:10.1177/0269881120954052

Everett NA, Turner AJ, Costa PA, Baracz SJ, Cornish JL. The vagus nerve mediates the suppressing effects of peripherally administered oxytocin on methamphetamine self-administration and seeking in rats. *Neuropsychopharmacology*. 2021;46(2):297-304. doi:10.1038/s41386-020-0719-7

Ezard N, Clifford B, Siefried KJ, et al. Lisdexamfetamine in the treatment of methamphetamine dependence: A randomised, placebo-controlled trial. *Addiction*. 2025;120(7):1345-1359. doi:10.1111/add.16730

Farahzadi MH, Moazen-Zadeh E, Razaghi E, Zarrindast MR, Bidaki R, Akhondzadeh S. Riluzole for treatment of men with methamphetamine dependence: A randomized, double-blind, placebo-controlled clinical trial. *J Psychopharmacol*. 2019;33(3):305-315. doi:10.1177/0269881118817166

Grant JE, Odlaug BL, Kim SW. A double-blind, placebo-controlled study of N-acetyl cysteine plus naltrexone for methamphetamine dependence. *Eur Neuropsychopharmacol*. 2010;20(11):823-828. doi:10.1016/j.euroneuro.2010.06.018

Heinzerling KG, Briones M, Thames AD, et al. Randomized, Placebo-Controlled Trial of Targeting Neuroinflammation with Ibudilast to Treat Methamphetamine Use Disorder. *J Neuroimmune Pharmacol*. 2020;15(2):238-248. doi:10.1007/s11481-019-09883-w

Heinzerling KG, Shoptaw S, Peck JA, et al. Randomized, placebo-controlled trial of baclofen and gabapentin for the treatment of methamphetamine dependence. *Drug Alcohol Depend*. 2006;85(3):177-184. doi:10.1016/j.drugalcdep.2006.03.019

Jeong H, Yoon S, Sung YH, et al. Effects of cytidine-5'-diphosphate choline on gray matter volumes in methamphetamine-dependent patients: A randomized, double-blind, placebo-controlled study. *J Psychiatr Res*. 2021;143:215-221. doi:10.1016/j.jpsychires.2021.09.006

Johnson BA, Ait-Daoud N, Elkashef AM, et al. A preliminary randomized, double-blind, placebo-controlled study of the safety and efficacy of ondansetron in the treatment of methamphetamine dependence. *Int J Neuropsychopharmacol*. 2008;11(1):1-14. doi:10.1017/S1461145707007778

Kangiser MM, Dwoskin LP, Zheng G, Crooks PA, Stairs DJ. Varenicline and GZ-793A differentially decrease methamphetamine self-administration under a multiple schedule of reinforcement in rats. *Behav Pharmacol*. 2018;29(1):87-97. doi:10.1097/FBP.0000000000000340

Kheirabadi GR, Bahrami M, Shariat A, Tarrahi M. The Effect of Add-on Buprenorphine to Matrix Program in Reduction of Craving and Relapse Among People With Methamphetamine Use Disorder: A Randomized Controlled Trial. *J Clin Psychopharmacol*. 2021;41(1):45-48. doi:10.1097/JCP.0000000000001320

Kheirabadi GR, Ghavami M, Maracy MR, Salehi M, Sharbafchi MR. Effect of add-on valproate on craving in methamphetamine depended patients: A randomized trial. *Adv Biomed Res*. 2016;5:149. Published 2016 Aug 30. doi:10.4103/2277-9175.187404

Ling W, Chang L, Hillhouse M, et al. Sustained-release methylphenidate in a randomized trial of treatment of methamphetamine use disorder. *Addiction*. 2014;109(9):1489-1500. doi:10.1111/add.12608

Ling W, Shoptaw S, Hillhouse M, et al. Double-blind placebo-controlled evaluation of the PROMETA™ protocol for methamphetamine dependence. *Addiction*. 2012;107(2):361-369. doi:10.1111/j.1360-0443.2011.03619.x

Mousavi SG, Sharbafchi MR, Salehi M, Peykanpour M, Karimian Sichani N, Maracy M. The efficacy of N-acetylcysteine in the treatment of methamphetamine dependence: a double-blind controlled, crossover study. *Arch Iran Med*. 2015;18(1):28-33.

Piasecki MP, Steinagel GM, Thienhaus OJ, Kohlenberg BS. An exploratory study: the use of paroxetine for methamphetamine craving. *J Psychoactive Drugs*. 2002;34(3):301-304. doi:10.1080/02791072.2002.10399967

Pittenger ST, Barrett ST, Chou S, Bevins RA. The effects of varenicline on methamphetamine self-administration and drug-primed reinstatement in female rats. *Behav Brain Res*. 2016;300:150-159. doi:10.1016/j.bbr.2015.11.033

Pittenger ST, Barrett ST, Chou S, Bevins RA. The effects of varenicline on methamphetamine self-administration and drug-primed reinstatement in male rats. *Behav Brain Res*. 2017;320:195-199. doi:10.1016/j.bbr.2016.12.005

Rezaei F, Emami M, Zahed S, Morabbi MJ, Farahzadi M, Akhondzadeh S. Sustained-release methylphenidate in methamphetamine dependence treatment: a double-blind and placebo-controlled trial. *Daru*. 2015;23(1):2. Published 2015 Jan 15. doi:10.1186/s40199-015-0092-y

Rezaei F, Ghaderi E, Mardani R, Hamidi S, Hassanzadeh K. Topiramate for the management of methamphetamine dependence: a pilot randomized, double-blind, placebo-controlled trial. *Fundam Clin Pharmacol*. 2016;30(3):282-289. doi:10.1111/fcp.12178

Salehi M, Emadossadat A, Kheirabadi GR, Maracy MR, Sharbafchi MR. The Effect of Buprenorphine on Methamphetamine Cravings. *J Clin Psychopharmacol*. 2015;35(6):724-727. doi:10.1097/JCP.0000000000000408

Schindler CW, Gilman JP, Panlilio LV, McCann DJ, Goldberg SR. Comparison of the effects of methamphetamine, bupropion, and methylphenidate on the self-administration of methamphetamine by rhesus monkeys. *Exp Clin Psychopharmacol*. 2011;19(1):1-10. doi:10.1037/a0022432

Schwienteck KL, Banks ML. Effects of 7-day continuous D-amphetamine, methylphenidate, and cocaine treatment on choice between methamphetamine and food in male rhesus monkeys. *Drug Alcohol Depend*. 2015;155:16-23. doi:10.1016/j.drugalcdep.2015.08.022

Shoptaw S, Huber A, Peck J, et al. Randomized, placebo-controlled trial of sertraline and contingency management for the treatment of methamphetamine dependence. *Drug Alcohol Depend*. 2006;85(1):12-18. doi:10.1016/j.drugalcdep.2006.03.005

Snider SE, Hendrick ES, Beardsley PM. Glial cell modulators attenuate methamphetamine self-administration in the rat. *Eur J Pharmacol*. 2013;701(1-3):124-130. doi:10.1016/j.ejphar.2013.01.016

Stauffer CS, Moschetto JM, McKernan S, et al. Oxytocin-enhanced group therapy for methamphetamine use disorder: Randomized controlled trial. *J Subst Abuse Treat*. 2020;116:108059. doi:10.1016/j.jsat.2020.108059

Stoops WW, Pike E, Hays LR, Glaser PE, Rush CR. Naltrexone and bupropion, alone or combined, do not alter the reinforcing effects of intranasal methamphetamine. *Pharmacol Biochem Behav*. 2015;129:45-50. doi:10.1016/j.pbb.2014.11.018

Trivedi MH, Walker R, Ling W, et al. Bupropion and Naltrexone in Methamphetamine Use Disorder. *N Engl J Med*. 2021;384(2):140-153. doi:10.1056/NEJMoa2020214

Wang F, Liu H, Ke Y, et al. Ibudilast-Mediated Suppression of Neuronal TLR4 in the Prefrontal Cortex Mitigates Methamphetamine-Induced Neuroinflammation and Addictive Behaviours. *Addict Biol*. 2025;30(4):e70033. doi:10.1111/adb.70033

Wang F, Shen W, Cai Y, et al. Buprenorphine reduces methamphetamine intake and drug seeking behavior *via* activating nociceptin/orphanin FQ peptide receptor in rats. *Front Psychiatry*. 2022;13:983595. Published 2022 Oct 6. doi:10.3389/fpsyt.2022.983595

Westenbroek C, Perry AN, Jagannathan L, Becker JB. Effect of social housing and oxytocin on the motivation to self-administer methamphetamine in female rats. *Physiol Behav*. 2019;203:10-17. doi:10.1016/j.physbeh.2017.10.020

Yoon SJ, Lyoo IK, Kim HJ, et al. Neurochemical alterations in methamphetamine-dependent patients treated with cytidine-5'-diphosphate choline: a longitudinal proton magnetic resonance spectroscopy study. *Neuropsychopharmacology*. 2010;35(5):1165-1173. doi:10.1038/npp.2009.221
